# The emotional impact on patients of venous thromboembolism

**DOI:** 10.1590/1677-5449.202201511

**Published:** 2023-02-10

**Authors:** Alcides José Araújo Ribeiro, Marcos Arêas Marques

**Affiliations:** 1 Hospital de Base do Distrito Federal, Brasília, DF, Brasil.; 2 Clínica de Veias, Brasília, DF, Brasil.; 3 Universidade Estadual do Rio de Janeiro – UERJ, Rio de Janeiro, RJ, Brasil.; 4 Universidade Federal do Estado do Rio de Janeiro – UNIRIO, Rio de Janeiro, RJ, Brasil.

Communicating a diagnosis of certain diseases, such as venous thromboembolism (VTE), can pose a major challenge. Since the emergence of the pandemic caused by Covid-19 and the publicity given to its association with VTE and death, patients’ fears and apprehension linked to the word “thrombosis” have increased greatly. There is a certain incompatibility between the physician’s objectives and the needs and anxieties of the patient who has suffered a VTE. In general, little attention is paid to emotional issues, but they rise to the surface when patients and their families are informed of the diagnosis.^1^

The psychological and emotional impacts on patients are rarely assessed in studies investigating VTE. Feehan et al.^2^ analyzed the psychological consequences for 907 patients with VTE, finding that around 40% of the study participants had daily fears of repeat thrombosis, 24.7% had abnormal levels of anxiety, and 11.6% had abnormal levels of depression. Younger patients reported greater VTE impact on their lives, with high levels of anxiety, fear, panic, nightmares, and symptoms of posttraumatic stress,^3^ while patients with VTE may have higher levels of anxiety than patients with other serious diseases, such as acute myocardial infarction.^2^

A Canadian study identified seven major themes related to patients’ experiences with VTE: acute impacts (initial shock and physical symptoms), sustained psychological distress (fear of recurrence and death), loss of self (changing habits and taking decisions for the future), challenges of VTE treatment (anticoagulation and elastic compression stockings), balance of life changes, negative experience with the medical system (delays and diagnostic errors), and VTE in the context of other conditions.^4^

Alarmist language and misplaced medical metaphors were identified as sources of anxiety in this context. Articles report phrases selected by patients as being highly harmful, such as: “you could have died if we did not make this diagnosis today”, “I’ve seen patients die from what you have”, and “you are a walking time bomb”.^5^ The physician should always use basic, layman’s language, at times and in places that are appropriate for communicating with the patient, using techniques for communicating bad news, such as the SPIKES protocol (setting up, perception, invitation, knowledge, emotions, strategy, and summary), for example. Constant checks should be made to ensure the patient understands every element covered and care must be taken with posture and non-verbal language, avoiding facial expressions of worry, tension, and indifference.^5^ Terms such as “postthrombotic panic syndrome” and “thromboneurosis” are increasingly common in the specialist literature.^6^

Physicians may contribute to postthrombotic psychological suffering, but they also can, and should, work to recognize and attenuate these manifestations, taking a range of actions, such as: ensuring early assessment by a specialist; actively listening to patients; dealing with their concerns; and providing information in hard copy and online about the disease and guidance about support groups, trustworthy websites, and specific keywords for searching the internet; in addition to employing educational resources, such as anatomic models, for example.^7^

The psychosocial impact of VTE can be highly traumatic, changing the patient’s life. Therefore, there is a real need to produce guidelines based on evidence specific to the management of these emotional sequelae and to implement programs for continuing medical education on the subject. Through recognition, dedication, and empathy, we can improve the experience of patients who have been the victims of VTE and contribute to their full recovery.

However, one question remains: “Do I communicate venous thrombosis cases to my patients in the best way possible?”.
