# Supplementary material for: The emotional impact on patients of venous thromboembolism
Source: J Vasc Bras. 2023 Feb 10;22:e20220151. doi: 10.1590/1677-5449.202201511 (PMC9925058; doi:10.1590/1677-5449.202201511)
Supplement: Translation

# Translation: O impacto emocional do paciente no tromboembolismo venoso

**DOI:** 10.1590/1677-5449.202201512

**Authors:** Alcides José Araújo Ribeiro, Marcos Arêas Marques

**Affiliations:** 1 Hospital de Base do Distrito Federal, Brasília, DF, Brasil.; 2 Clínica de Veias, Brasília, DF, Brasil.; 3 Universidade Estadual do Rio de Janeiro – UERJ, Rio de Janeiro, RJ, Brasil.; 4 Universidade Federal do Estado do Rio de Janeiro – UNIRIO, Rio de Janeiro, RJ, Brasil.

A comunicação do diagnóstico de certas doenças, como o tromboembolismo venoso (TEV), pode ser um grande desafio. Com o advento da pandemia causada pela covid-19 e a divulgação da sua associação com TEV e morte, o medo e a apreensão dos pacientes com a palavra “trombose” aumentaram muito. Existe uma certa incompatibilidade entre os objetivos do médico e as necessidades e os anseios do paciente vítima de TEV. Em geral, dedica-se pouca atenção às questões emocionais, e estas afloram substancialmente ao informar um paciente e sua família sobre esse diagnóstico^1^.

Os impactos psicológicos e emocionais nesses pacientes raramente são avaliados nos estudos relativos ao TEV. Feehan et al.^2^, ao analisarem as consequências psicológicas em 907 pacientes com TEV, encontraram medo diário de retrombose em torno de 40% dos participantes, quadro de ansiedade em 24,7% e quadro de depressão em 11,6%. Os pacientes mais jovens relatam um impacto maior do TEV em suas vidas, com altos níveis de ansiedade, medo, pânico, pesadelos e sintomas de estresse pós-traumático3, e pacientes com TEV podem ter mais ansiedade do que pacientes com outras doenças graves, como infarto agudo do miocárdio^2^.

Um estudo canadense levantou sete temas maiores relacionados à experiência do paciente com TEV: o impacto agudo (choque inicial e sintomas físicos), o sofrimento psicológico persistente (medo de recorrência e morte), a perda de si mesmo (mudanças de hábitos e tomada de decisões para o futuro), os desafios no tratamento do TEV (anticoagulação e meias de compressão elástica), o equilíbrio nas mudanças de vida, a experiência negativa com o sistema de saúde (atrasos e erros diagnósticos) e o TEV no contexto de outras doenças^4^.

A linguagem alarmista e as metáforas médicas mal colocadas foram identificadas como fontes de ansiedade neste contexto. Artigos relatam frases selecionadas pelos pacientes como altamente prejudiciais, como: “já vi pacientes morrerem com o que você tem”, “você poderia ter morrido se não tivéssemos feito este diagnóstico hoje” e “você é uma bomba-relógio ambulante”^5^. O médico deve sempre usar linguagem básica e leiga, com tempo e local adequados para se comunicar com o paciente, procurando usar técnicas de comunicação de más notícias, como o protocolo SPIKES (do inglês, *setting up, perception, invitation, knowledge, emotions, strategy and summary*), por exemplo. Deve-se verificar constantemente a compreensão do paciente sobre cada componente abordado e observar o cuidado com a postura e a linguagem não verbal, evitando fácies de preocupação, tensão e descaso^5^. Termos como “síndrome do pânico pós-trombose” e “tromboneurose” são cada vez mais frequentes na literatura especializada^6^.

Os médicos podem contribuir para o sofrimento psicológico pós-trombose, mas podem e devem atuar no sentido de reconhecer e atenuar essas manifestações através de várias ações, como: a avaliação precoce por um médico especialista, a escuta ativa do paciente, a abordagem de suas preocupações, o fornecimento de informações impressas e *on-line* sobre a doença e a orientação sobre grupos de apoio, sites confiáveis e palavras chaves específicas para pesquisa na internet, além de uso de recursos didáticos como modelos anatômicos, por exemplo^7^.

O impacto psicossocial do TEV pode ser muito traumático e mudar a vida do paciente; portanto, urge a necessidade de disponibilizar diretrizes baseadas em evidências específicas para o manejo dessas sequelas emocionais, bem como a criação de programas de educação médica continuada neste tópico. Com reconhecimento, empenho e empatia, podemos melhorar a experiência e a recuperação integral dos pacientes vítimas do TEV.

E fica o questionamento: “Eu comunico o caso de trombose venosa do meu paciente da maneira ideal?”.
